# Inverting the pyramid! Extent and quality of food advertised on Austrian television

**DOI:** 10.1186/s12889-015-2275-3

**Published:** 2015-09-18

**Authors:** Benjamin Missbach, Adelheid Weber, Elke M. Huber, Jürgen S. König

**Affiliations:** Department of Nutritional Sciences, University of Vienna, Althanstrasse 14, 1090 Vienna, Austria

**Keywords:** Advertising, Children, Television, EU Pledge Nutrition Criteria, Food marketing regulation, Nutrition profiling criteria

## Abstract

**Background:**

Research showed that food marketing for children frequently contradicts national dietary guidelines. Children, unlike adults, are not able to understand the persuasiveness of the advertisements with its short- and long-term effects on health, thus the common international tenor is to restrict food marketing. In the European Union, marketing restriction based on self-regulation have been initiated (EU Pledge Nutrition Criteria). The study aims contribute to depict the status quo of television advertisement targeted at children before the pledged initiative came into full effect.

**Methods:**

In this study we analyze the quality and displaying frequency of a set of advertisements targeted at children broadcasted on Austrian television. Promoted food products targeted at children or adults were identified. Category-based analysis of the displayed food was performed based on the Austrian Nutrition guidelines (number of displayed food per food category). The children’s food content was analyzed according to the newly established nutritional quality criteria for advertised food in the EU to assess the nutritional quality of the depicted food.

**Results:**

In total, 360 h of video material was recorded in February and March 2014. A set of 1919 food advertisements, with 15.1 % targeted at children were broadcasted. Of all food advertisements targeted at children, 92.4 % was for fatty, sweet and salty snacks, while no advertisements for vegetables, legumes or fruits were shown. From all food advertisements for children, 65.9 % originated from participating companies of the EU Pledge Nutrition Criteria. Further analysis revealed that 95.9 % of the advertised food for children showed at least one aspect of nonconformity with the EU Pledge Nutrition Criteria; on the contrary 64.7 % of the displayed food advertisement also featured at least one desirable food component (e.g. high fibre content, high protein content).

**Conclusions:**

The present research suggests that the majority of advertised food for children do not conform with the pledged criteria as defined in the EU Pledge Nutrition Criteria and almost all advertisements would be prohibited. We discuss our findings in the context of public health nutrition and present a perspective for future directions in this important field of research.

**Electronic supplementary material:**

The online version of this article (doi:10.1186/s12889-015-2275-3) contains supplementary material, which is available to authorized users.

## Background

Food marketing holds double-edged characteristics. Marketing practices, such as the promotion of potentially unhealthy food as well as the promotion of potentially healthy food, can increase consumption both in children and adults [1, 2]. Research on advertisements targeted at children has shown that unhealthy food, respectively energy-dense, high-fat and sugary foods are more often advertised in television (TV) in spite of first marketing restrictions inititiated over a decade ago [3]. This marketing component shapes modern obesogenic environments. Increased accessibility and salience in conjunction with a sedentary lifestyle are important drivers in the global obesity pandemic [4, 5]. In fact, a recent meta-analysis by Chapman et al. [6] showed that one of the three most prominent lifestyle factors for increased short-term effects on food intake is watching TV. The effect of TV on eating habits was shown in both laboratory [7] and epidemiological studies [8, 9]. Interventions to restrict unhealthy as well as to promote healthy food marketing targeted at children, is a major public health effort to promote eating habits [10]. The most challenging issue however, is to unambigously define what is unhealthy [11], as the establishment of applied models of choice will have considerable influence on the outcome of food marketing regulations [12].

The present study aims to identify the extent of food marketing and the nutritional quality of the advertised food targeted at children on Austrian TV. To assess the current status quo of food advertisements for children, we analyze the food quality based on the suggested guidelines for nutritional criteria in the European Union (EU Pledge Nutrition Criteria). We analyze advertisement data before the guidelines came into force (prior to the 31st of December 2014) to set a starting point for future analysis of the potential impact of the EU Pledge Nutrition Criteria on food advertisement targeted at children.

To date, there is no study analyzing the Austrian TV landscape based on the recently established criteria and only a few international studies describe the status quo before the voluntary commitment for the new criteria came into force.

## Food marketing targeted at children

Food marketing is intended to have persuasive effects on children’s food preferences, food purchasing behavior and short-term food consumption [13, 14]. For instance, children who watched more TV responded to frequently advertised food items more readily than those who watched less [15, 16]. Even brief exposure to food advertising influenced children’s food preferences [17]. As shown by Harris, Bargh and Brownell [1], elementary-school-aged children consumed 45 % more snacks when exposed to food advertisements. In adults, this effect was dissociated of the participants’ reported hunger, indicating that this particular snacking behavior is guided by automatic and habitual processes [18]. Repetitive exposure to food products as food primes can enhance the effect on shaping eating habits (mere-exposure effect [19]) through the Pavlovian stimulus–outcome (S-O) conditioning, therefore eliciting short-term food cravings [20].

Children as young as three years old are capable to identify advertisements during TV programs but, unlike adults, are not able to understand the persuasiveness of advertisements and their short- and long-term effects on health [21]. This makes food marketing targeted at children a deceptive way to propose commercial transactions to children and should therefore be restricted [22]. Consquently, international authoritites, including World Health Organization (WHO) member states, called for a general agreement to harmonize food and beverage advertisements within national dietary guidelines of the Global Strategy on Diet, Physical Activity and Health (2004). A systematic review by Galbraith-Emami and Lobstein [23] showed that worldwide initiatives are effective in restricting advertisement on-air time for children, but the effectiveness depends highly on audience definition and nutrient profile criteria. In some countries, limiting advertisement of energy-dense, nutrient-poor food and beverages is implemented in their national action plans on nutrition [24]. Inspite of measures taken, global food marketing, especially unhealthy food for children, appear to be on the rise since first regulatory actions have been initiated [25, 26].

## The EU Pledge Nutrition Criteria

In the absence of complete bans of food advertisements, there is a need aimed at a comprehensive and consistent approach across countries to regulate the quantity and quality of the advertised content. International regulatory guidelines have engaged different models, such as nutrient or category-based approaches. In Europe, the EU Pledge Nutrition Criteria are conceptualized to restrict food and beverage advertisements to children younger than 12 years old on TV, print media and on the internet [27]. It is designed as a self-regulatory and voluntary intitative including 20 companies being responsible for at least 80 % of the TV food marketing in the EU. The self-regulatory pledged criteria use a dual strategy to encourage the advertisement of healthy food components (e.g. high fiber and protein content) and simultaneously aim to restrict unhealthy food components (e.g. high amounts of sugar, saturated fatty acids). The voluntary commitment started in 2012 and participating members pledged to implement the suggested guidelines by 31st December 2014.

The EU Pledge Nutrition Criteria use 9 different food categories to arrange food advertisements. In short, food groups, such as soft drinks, or sugar and sugar-based products, are fully restricted for advertisement. Food products that promote a particular food within a certain category have to contain category-specific components per defintion (e.g. for dairy products to contain at least 50 % dairy), otherwise they should not be advertised. Additionally, depending on the presented food category, nutrient-based thresholds are defined for energy (kcal/portion), sodium (mg/100 g or 100 ml), saturated fats (mg/100 g or 100 ml) and total sugars (g/100 g or 100 ml).

Additional to nutrient-based thresholds, certain food components should be encouraged (nutrients and food groups). Desirable food components are qualified as positive and should be encouraged: such as fibre and whole grain in cereal-based products; protein and calcium in milk and dairy products; protein in meat, fishery products; polyunsaturated fatty acids (PUFAs) in vegetable oils, spreads and fats.

## The current study

The present study was conducted before the EU Pledge Nutrition Criteria came into full effect (prior to 12/2014) to provide a status quo analysis regarding food advertisements aired on Austrian TV. In detail, we present data from advertisements, displaying frequency and the nutritional quality of the advertised food products. The aim of this study is threefold: we (i) analyze a set of advertisements broadcasted for children on six different TV stations. We split the advertised food into eight food categories based on the Austrian dietary guidelines as defined by the Austrian Food Guide Pyramid [28]; (ii) we provide data regarding the nutritional quality of food advertisements targeted at children by comparing the displayed food with the pledged EU Nutrition Criteria, and (iii) we discuss the results and their implications in a broader context of public health nutrition.

## Methods

We used a two-step approach to collect data in this study. First, we recorded and analyzed TV programs. We applied the national dietary guidelines to develop a coding scheme. Two coders identified advertisement orientation (children vs. adults) and target food (displayed food categories). In the second step, the first and second author of the study provided a closeup of the quality of the food advertisements and analyzed the advertised products according to the EU Pledge Nutrition Criteria. As this study did not involve human subjects, *The Code of Ethics of the World Medical Association* for experiments did not apply and the University of Vienna Ethics Committee granted a waiver for ethical approval.

## Data collection

Data collection took place in February and March 2014. TV programs were recorded from six seperate TV channels over four days (two non-consecutive weekdays and two independent days on the weekend). TV channels were selected based on media analysis of children’s TV habits in Austria, Germany and Switzerland [29], choosing the six most popular TV channels for children (ATV, ORF1, Pro7, RTL, Sat1 and SuperRTL). Recording period for each dedicated day was from 6 am to 9 pm. Recording time was further split into three time periods: morning hours (6 am – 11 am), midday hours (11 am – 4 pm) and evening hours (4 pm – 9 pm).

In total, 360 h of broadcast material was recorded, spread equally over all six TV stations. We recorded full days of TV screening (6 am – 9 pm) to get a complete and consistent dataset of recordings. Previous studies reported segmented recording times (e.g. morning, after-school hours) [30] or solely recorded kids programs [31, 32] which may have led to incomplete datasets for the analysis. In this study, full days from 6 am to 9 pm and all types of programs were recorded to reduce the chance of missing any data. All stations broadcasted their program in German.

## Review and coding reliabilty of TV advertisements

First, the content of the TV program was divided into non-programs and programs (e.g. TV shows, news updates). Non-programs included promotion of station programs, station identification, and product advertisements. To identify advertisements, we used a coding protocol established by Thompson et al. [33]. Brief sponsorship messages, such as “this program was brought to you by product X”, brand display or buying recommendations, were used to identify advertisements [33]. Hence, product advertisements were divided into food or non-food advertisements. As we only focus on food advertisements in this study, we did not record the percentage of food advertisements compared to non-food advertisements. Food advertisements were considered as such when they promoted the purchase or consumption of food or beverages. In a second step, we identified if food advertisements were targeted at children. This was conducted according to a dichotomous categorization scheme adapted from Chapman et al. [34] (Table 1).

**Table 1:** Criteria to determine advertisement orientation (target audience) and displayed food categories (target food categories)

Target audience (children)	Target audience (adults)
Animation	Adults or adult celebrities
Children or child celebrities	Adult-oriented music
Pets or animals	Adult-focused voice or dialogue
Identifiable cartoon characters, mascots, promotion of fun	
Child-focused music	
Child-friendly voice or dialogue	
English words and expressions	
Children singing	

Adapted from Chapman et al. 2006 [34]

The video material was reviewed by two coders fluent in German (author 2 and 3). Initial coding of the video material was performed by one coder, a second coder was given the same coding form and instructions to code a 10 % sample of the total duration of recordings (36 h). Inter-coder reliability was calculated using the following formula: number of agreements*100 / number of disagreements. This reliability check was only performed for audience orientation and not for the food categorization procedure.

Inter-coder reliability was 95.8 % in the present study. All discrepancies emerging during the process were discussed within the research team.

## Advertised food categories

Initially, to identify food categories depicted in the advertisements, we categorized food according to the national dietary recommendations. The Austrian Food Guide Pyramid is described in seven categories [28]. According to the recommendations, items from each category should be eaten at different frequencies per day (Table 2). In addition, we added one extra category called ‘other food not further specified’ to complement the categories of the Austrian Food Guide Pyramid. All discrepancies emerging during the categorization process were discussed within the research team and conflicting food categorizations were resolved in consensus. Hence, we analyzed the video material according to following eight food categories:

**Table 2:** Categorization code used to determine displayed food categories by means of the Austrian Food Guide Pyramid

Target food category	Consumption recommendation according to the Austrian Food Guide Pyramid
Category 1: Fatty, sweet and salty snacks	Sweets, pastries, fast food products, snacks, munchies and soft drinks are nutritionally less recommended and should be consumed rarely – a maximum of one serving per day. Avoid heavily salted foods e.g. pickled foods, snacks, salted nuts, convenience products
Category 2: Fats and vegetable oils	1–2 tablespoons of vegetable oils, nuts and seeds daily. High quality vegetable oils such as olive oil, canola oil, walnut, soybean, linseed, and nuts, and also seeds contain essential fatty acids an can be consumed daily in a moderate amount (1–2 tablespoons). Other fats such as butter, margarine and lard and several fatty dairy products (e.g. whipped cream, sour cream and crème fraîche) should be used sparingly.
Category 3: Fish, meat, sausages and eggs	Eat at least 1–2 servings of fish (each approx 150 g) per week and prefer fatty sea fish (mackerel, salmon, tuna and herring) or local cold water fish such as char. Eat a maximum 3 servings of lean meat or low-fat sausages (300–450 g /week) per week. Eat red meat (such as beef, pork and lamb) and sausages rarely. Up to 3 eggs can be consumed per week.
Category 4: Milk and dairy products	Consume 3 servings of milk and dairy products each day. Prefer low fat alternatives. 1 serving equals: milk (200 ml), yogurt (180–250 g) cottage cheese (200 g), curd cheese (200 g), cheese (50–60 g).
Category 5: Cereal products and potatoes	Eat 4 servings of cereals, bread, pasta, rice or potatoes. 1 serving equals: whole wheat bread (50–70 g), buns and bagels (50–70 g), cereals (50–60 g), pasta (uncooked 65–80 g, cooked 200–250 g), rice or corn (uncooked 50–60 g, cooked 150–180 g), potatoes (cooked 200–250 g). Prefer whole grain products.
Category 6: Vegetables, legumes and fruits	Eat 5 servings of vegetables, legumes and fruits per day. 3 servings of vegetables and legumes and 2 servings of fruit would be idea. 1 serving equals: vegetables (cooked 200–300 g, raw 100–200 g), salad (75–100 g), legumes (cooked 150–200 g, raw 70–100 g), vegetable or fruit juice (200 ml).
Category 7: non-alcoholic beverages (e.g. water, tea, coffee)	Drink at least 1.5 l of liquids per day, prefer low-energy beverages (e.g. tap water, mineral water, unsweetened teas and diluted fruit or vegetable juices). A daily moderate consumption of coffee, black tea (3–4 cups) and other caffeinated beverages is acceptable.
Category 8: other food not further specified	miscellaneous e.g. mixed dishes, baby food, convenience products

Adapted form the Austrian Food Guide Pyramid [28]

- Fatty, sweet and salty snacks (e.g. cakes, fast-food products, chips)
- Fats and vegetable oils (e.g. olive oil, nuts, butter)
- Fish, meat, sausages and eggs (e.g. tuna, salami, processed meat)
- Milk and dairy products (e.g. yogurt, cheese, milk)
- Cereal products and potatoes (e.g. bread, granola, rice)
- Vegetables, legumes and fruits (e.g. beans, salad, tomatoes)
- non-alcoholic beverages (e.g. water, tea, coffee)
- other food not further specified (e.g. convenience food, baby food)

We assessed the on-air frequency of the advertisements by counting the total number of advertisements. We interposed this step to analyze the dataset according to the Austrian Food Guide Pyramid to assess how the foods depicted on TV match with the national dietary recommendations.

In a second step, the displayed products were analyzed according to the EU Pledge Nutrition Criteria. This was conducted to assess how the foods depicted on TV match with the EU Pledge Nutrition Criteria. According to the EU Pledge Nutrition Criteria 9 different food categories are defined. For this we re-coded the displayed advertisements accordingly and conducted nutrient profiling analysis with those foods initially passing the category criteria (exclusion criteria: food groups such as soft drinks, sugar and sugar-based products and misleadingly declarated foods; see supplementary material, Additional file 1).

For further analysis, the nutritional information of the food products was obtained directly from the nutrition information on the label of the promoted products during supermarket visits or online. When nutrition information was not readily available, manufacturers were contacted. The data was then compared with the nutrient threshold limits and the food components that should be encouraged as defined by the EU Pledge Nutrition Criteria by the first and second author of this study.

## Statistical analysis

Statistical analyses were conducted using IBM SPSS 22. Descriptive statistics were used to explore the frequency of displayed TV food advertisements, while chi-square tests were used to compare the proportion of displayed food categories by target audience and displaying frequency on different times of the day. Results were considered significant at an α level of *p* ≤ 0.05.

## Results

### Overview

**Fig. 1:**
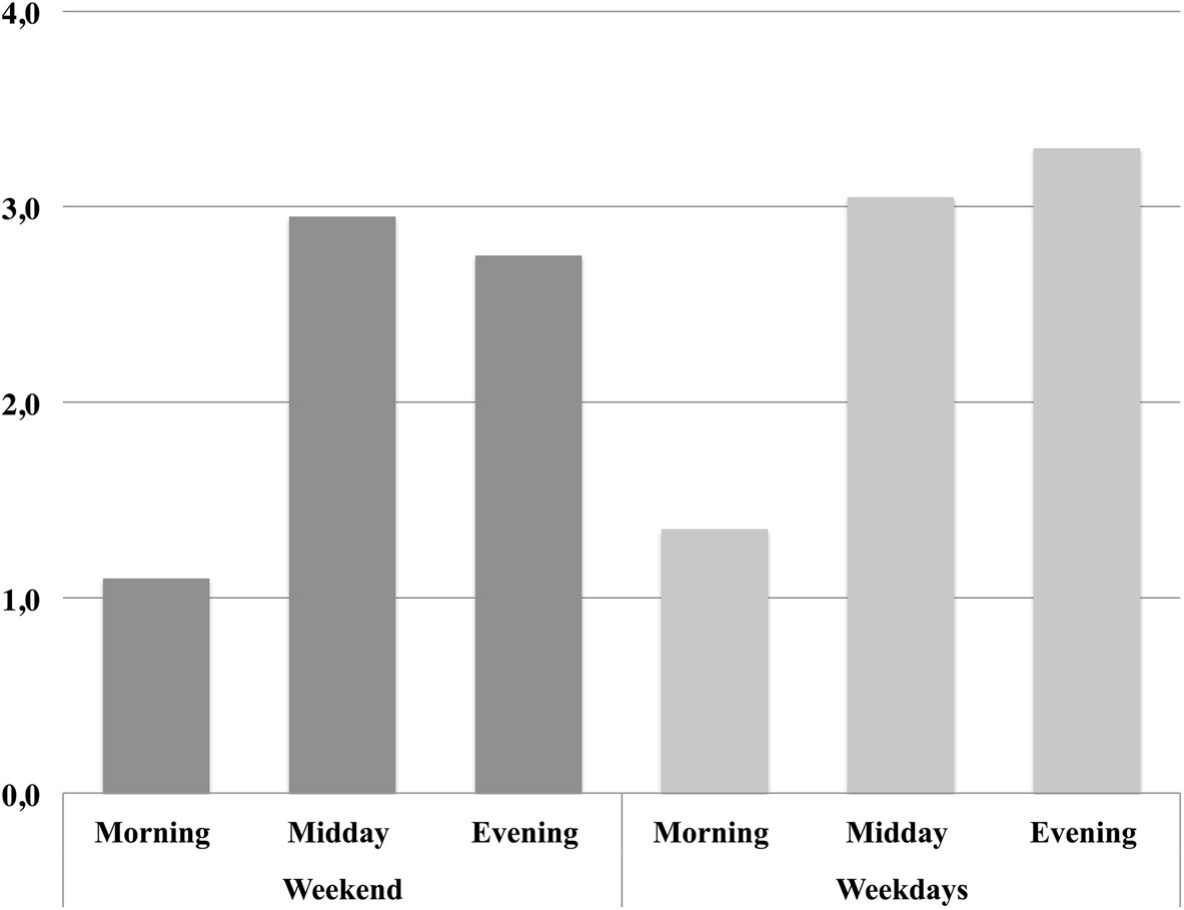
Displaying frequencies of food advertisements for children (per hour). *Notes*. Advertisements are displayed in displaying frequencies per hour on different times of the day, split into weekends and weekdays. Morning hours (6 am–11 am), midday hours (11 am–4 pm), evening hours (4 pm–9 pm)

A total of 1919 food advertisements were displayed in 360 h of recorded video material. Most food advertisements were shown between 11 am and 4 pm (*n* = 734), compared to evening hours from 4 pm to 9 pm (*n* = 719) and morning hours from 6 am to 11 am (*n* = 466). For the single food advertisement, the average air time was 25.05 s (standard deviation: ± 7.97 s). Within the complete recording period, 290 food advertisements targeted at children were identified (15.1 %) and 1629 advertisements were targeted at adults (84.9 %). For children, there was no difference in advertisement frequency on weekends compared to week days (*χ*^2^(1) = 1.08, *p* > .05). Food advertisement displaying frequency did differ significantly comparing morning, midday and evening blocks, *χ*^2^(2) = 10.20, *p* < .05 (see Fig. 1). In detail, food advertisements for children were less frequent during morning hours compared to midday hours, *χ*^2^(1) = 10.13, *p* < .05 but not compared to evening hours, *χ*^2^(1) = 2.62, *p* > .05. Most advertisements for children were shown during evening hours (*n* = 121).

### Advertised food categories

**Fig. 2:**
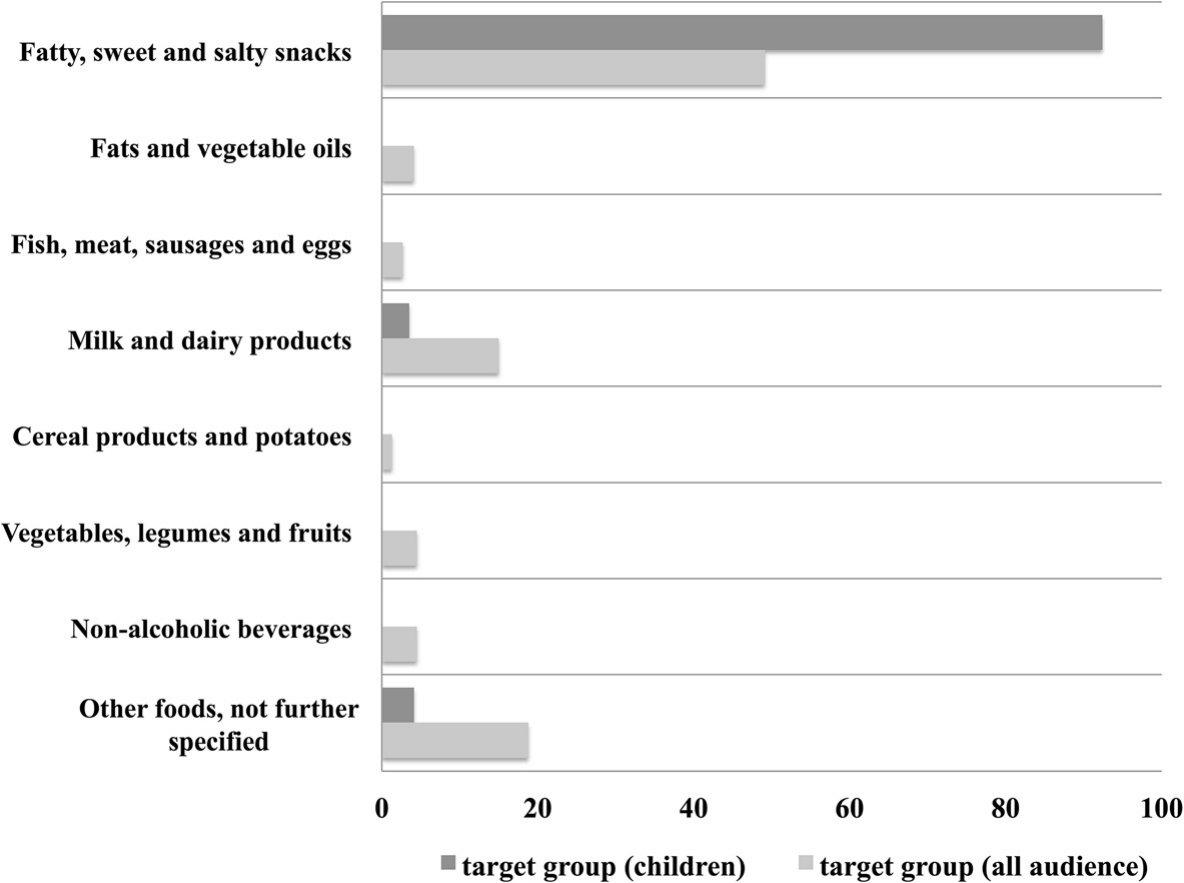
Displaying frequency of eight food categories divided by target group (in %). *Notes*. Displaying frequency of eight food categories of the Food Guide Pyramid in % of the total displayed food. The displaying frequency is divided along the target group, respectively targeted at children contrasted by all audiences

Of all displayed 1919 food advertisements (targeted at children and adults), a total of 161 food products from 72 different companies were aired. In our sample, we identified three main companies that provided food advertisements: Ferrero Inc. (24.1 %), Danone Inc. (9.7 %) and Unilever Inc. (7.9 %). According to the Austrian Food Guide Pyramid, 49.1 % of all the displayed food were for fatty, sweet and salty snacks, 18.8 % for convenience food and 15 % for milk and dairy products. Displaying frequency of vegetables, legumes and fruits and non-alcoholic beverages was 4.5 %, while 4.1 % of the food advertisements were for fats and vegetable oils. Fish, meat, sausages, and eggs were addressed in 2.7 % of the food advertisements and cereal products and potatoes were displayed the least frequent (1.3 %) (Fig. 2).

For children, most commonly advertised food categories were fatty, sweet and salty snacks (92.4 %), convenience food (4.1 %) and milk and dairy products (3.5 %). All other food categories (fats and vegetable oils; fish, meat, sausages and eggs; vegetables, legumes and fruits; cereal products and potatoes and non-alcoholic beverages) were not advertised for children in our sample. There was a significant association between the displaying frequency of fatty, sweet and salty snacks and whether advertisement were targeted at children or adults *χ*^2^(1) = 255.97, *p* < .001. Based on the odds ratio, food advertisement for children for fatty, sweet and salty snacks was 17,2 times higher than for adults.

### Nutrition quality of the advertised food targeted at children

All 290 food advertisements targeted at children were included for further analysis against the EU Pledge Nutrition Criteria. The displayed advertisements were spread across 20 different products by 11 food companies. Almost 2/3 of the food advertisements for children originated from participating companies of the EU Pledge Nutrition Criteria (65.9 %). Food was shown at different displaying frequencies (ranging from 1 to 48 repetitions) over the recorded time frame.

Based on the category restrictions, 58.9 % of food advertisements did not pass the pledged criteria. In detail, 69 % were excluded, because they were declared as dairy products, but did not fulfill the corresponding criteria (e.g. dairy products must contain at least ≥ 50 % dairy); 15.2 % of the food advertisements promoted soft drinks, 13.5 % represented sugar or sugar-based products and 2.3 % represented combinations of fast food meals (see supplementary material, Additional file 1).

Advertisements passing the criteria for the corresponding category were used for an in-depth nutrient-based threshold analysis (41.1 % of the food advertised for children). Advertisements represented three EU Pledge Nutrition Criteria categories: EU Pledge Category 3: 14.3 % from meat based products; EU Pledge Category 5: 64.7 % from dairy products; EU Pledge Category 6: 21 % from sweet biscuits, fine bakery wares and other cereal based products.

At least one food component that should be encouraged in advertisements for children was identified in 64.7 % of the advertisements. As such, protein >12 E% or > 2 g /100 g or 100 ml was among most advertisements, as well as desirable trace elements (e.g. calcium) and micronutrients (e.g. vitamin D, vitamin B).

**Fig. 3:**
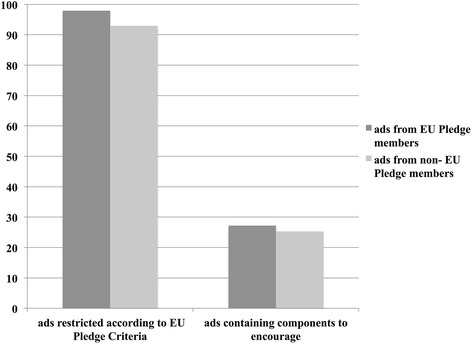
Food advertisement by member and non-member companies displaying encouraging and restricted foods (in %). *Notes*. Displaying frequency of food advertisements by members and non-members of the EU Nutritition Criteria Pledge split in % of restricted food advertisement and % of food advertisement displaying food components to encourage

On the contrary, from all advertised food targeted at children, in 95.9 % of the adversiements at least one characteristic was identified not in line with the EU Pledge Nutrition Criteria. 97.9 % of the advertised foods by participating companies of the EU Pledge Nutrition Criteria and 91.9 % of non-members failed to pass the criteria (Fig. 3). In total, only 10.1 % of the nutrient analyzed advertisements passed all criteria for nutrient-based threshold (see supplementary material, Additional file 2).

## Discussion

The results of this study include two main important findings. First, our data suggest that frequency of food advertising for children on Austrian TV can be attributed to three main food categories: fatty, sweet and salty snacks (92.4 %), convenience food (4.1 %) and dairy and dairy products (3.5 %). This distribution is absolutely in conflict to the present food intake recommendations provided by the Austrian Federal Ministry of Health. The findings of the study are in line with previous studies, showing a similar distortion between intended food recommendation and actual food advertisement patterns [30, 34–36]. For instance, Huang and colleagues (2012) showed that 46 % of food advertisements for children in Singapore were for candy, confectionery and fast food [31]. In contrast, advertisement for food associated with positive health effects (fruits, vegetables, whole grain foods, etc.) are nonexistent in our data.

The second major finding resulted from an in-depth analysis of the food contents marketed to children. Our dataset showed that 95.9 % of food advertisements would be restricted according to the newly established EU Pledge Nutrition Criteria. More than half of the food advertisements (58.9 %) did not pass the marketing criteria based on the category restrictions, and from the remaining 41.1 % only 10.1 % passed the criteria for nutrient threshold. In comparison, Scarborough and colleagues [12] reported that when applying the EU Pledge Nutrition Criteria to 336 food products, only 25.6 % would have been banned. The authors of the study investigated all food displayed during a one year time period [12]. In contrast, in our study we applied the restriction criteria to food especially targeted for children. Our approach may be more straightforward than applying the restriction model to all advertised food and more suitable for this particular research question. In fact, in line with our findings, Gunderson et al. [37] investigated food marketed on different TV stations for children and could show that 92.1 % of the food advertisements targeted at children was food associated with negative health effects.

Notwithstanding the presented data about the displaying frequency of food with negative health effects, our study also showed that 64.7 % of the advertisements targeted at children contain desirable food components according to EU Pledge Nutrition Criteria (e.g. high protein content, calcium, vitamin D, vitamin B). The pledge consortium’s reasoning for taking desirable food components into account was to foster innovation, reformulation and competition in the EU. The proclaimed goal of the pledged nutritional criteria is to shift advertising towards improved products [27]. However, by taking a closer look at the food that contain desirable components, almost all of them also contain nutrients to limit. This approach may be an advantage for producers to identify good or problematic formulations of their products. On the other hand, self-regulatory nutrient guidelines were shown to be ineffective in reducing unhealthy food advertisement targeted at children worldwide [38]. As most members of the EU Pledge Nutrition Criteria consortium are mutlinational companies, no pioneering efforts have been made so far [23]. Additionally, the time it takes until a reformulated food compliant with the pledged criteria becomes market-ready, may leave another generation of children watching TV with unhealthy food advertisement. Thus, the finding that 64.7 % of the advertisements targeted at children contain desirable food components according to the pledged nutritional criteria should be interpreted with caution.

## Public health perspective

In our study we show that one in seven food advertisement was targeted at children, with 92.4 % displaying fatty, sweet and salty snacks. In Austria, the average TV viewing time, from the age of three years on, is approximately 158 min per day [39]. TV watching in excess of 120 min and longer is associated with reduced physical and psychosocial health, and a large body of evidence suggests that especially decreasing sedentary time in youth aged 5–17 years leads to reductions in Body-Mass Index and health risks [40]. The reduction of sedentary lifestyles and promotion of early life nutritional education to strengthen overall self-regulatory resources may be key to responsible eating behavior. This has been addressed in a dual-model on a population-based childhood obesity prevention program provided by the WHO [41]. Regulating food marketing for children may be one effective measure to decrease food exposure to children [23]. Our results support the idea that food advertisement seem to distort national dietary recommendations, especially when the recipients of the advertisements are children (e.g. 17 times more likely for fatty, sweet and salty snacks). In Austria, for instance, five servings of fruits or vegetables per day are recommended, none of them were displayed in our study, although the National Action Plan for nutrition explicitly stresses the promotion of healthy food, such as fruits [42].

In the present study we investigated food advertisements by means of their eligibility within the EU Pledge Nutrition Criteria. There is a political alignment accompanied by voluntary codes of conduct by some food companies [43]. A preliminary and post-implementation study evaluating the self-regulatory Children’s Food and Beverage Advertising Initiative in Canada (2006–2011) came to the conclusion, that while the volume of aired advertisements for children decreased by 24 %, the advertisement quality decreased likewise. They showed that compared to 2006, food classified as less healthy were increasingly targeted at children (+47 %) and teenagers (+161 %) after implementing the initiative. The authors concluded that, in Canada, the initiated self-regulatory system designed to protect Canadian children from food advertising showed clear weaknesses and advertisement should be regulated more strictly [44]. A recent systematic review of evidence by Ronit and Jensen [45] provides an overview concerning research on industry self-regulation regarding food and beverage marketing and nutrition labeling. The authors conclude, that although methodological heterogenity was prevalent in the 22 reviewed articles, the ineffectiveness of existing self-regulation schemes is univocal, calling for more legislative guidelines. In addition, the use of commonly persuasive techniques, such as premium offers, promotional characters, nutrition and health-related claims, the theme of taste, and the emotional element of fun, are frequently displayed in the endorsement of food for children [46]. Especially promotional characters, like cartoon characters, have shown to influence food preferences in children, but branding has mainly been used for energy-dense and nutrient-poor foods (e.g. cookies, candy or chocolate), as compared to fruits or vegetables [47]. In the EU Pledge Nutrition Criteria, no restrictions for persuasive marketing techniques (mascots, cartoon characters) are implemented. Considering this and the findings of our study, that 65.9 % of the advertised food for children originate from member companies of the EU Pledge Nutrition Criteria and 97.9 % of their advertised foods showed nonconformity aspects, an argument for stricter regulations may be put forward.

As part of a new wave in public health improvements, the promotion of healthy food and nutrient components as proposed by the EU Pledge Nutrition Criteria would fit well into the new’cultural turn’ in the field of public health as proposed by Davies et al. [48]. Inarguably, to further minimize influences towards unhealthy behavior, it is necessary to both maximize the value of health and the promotion of healthy choices as default and to minimize factors that create an environment of unhealthy behaviors [49].

## Limitations

One major limitation of the present study is that we did not screen for seasonal differences and only presented data from a 2 month time frame (February and March 2014). Nevertheless, our results showed comparable results and displaying frequencies, which have been shown in other studies as well. A second limitation is more general and aimed at criticizing the definition of nutritional criteria. Different nutrient profiling strategies have been developed to ban advertisements targeted at children. Throughout this process, the question what is *healthy food* and what is not, proved to be difficult. To support this argument, Scarborough et al. [12] showed that when using eight different nutrient profile models on a dataset of food advertisements, the percentage of permitted food advertisements varied from 2.1 to 47.4 %. Although the authors used diverging models, the study shows that advertisment restraint highly depends on the applied restriction model. From this point of view, defining nutrient criteria as basis for marketing restrictions have to be grounded on solid scientific findings, taking all available evidence into consideration. In contrast, Lobstein and Davies [11] argued that nutrient profiling as a method to categorize food according to nutritional quality is both feasible and practical and can support a number of public health-related initiatives. A third limitation of the study is that we cannot provide data about causal effects of TV advertisements on food habits and eating behavior. Our study design is therefore limited to a mere descriptive level.

## Conclusions

In conclusion, we highlighted several important issues regarding food advertisement targeted at children and showed that in Austria, advertised food is not in accordance with the Austrian dietary guidelines and is mainly nonconfirmatory with the newly established EU-wide nutrition criteria for food advertisement targeted at children. This research provides a good starting point for future monitoring the success of the EU Pledge Nutrition Criteria. Based on our findings, procedures for continous and comprehensive monitoring for self-regulatory pledges in food marketing targeted at children are necessary. This field of research needs further investigation to pinpoint precise tools by which to restrict unhealthy food advertisements, and to promote healthy food in modern media environments.

## Additional files

Additional file 1:
**Identified food products displayed in food advertisement, category and classification according the the EU Pledge Nutrition Criteria.** (DOCX 101 kb) Additional file 2:
**Identified food products displayed in food advertisement, nutrient-based analysis according to the EU Pledge Nutrition Criteria.** (DOCX 36 kb)
