# Supplementary material for: Inverting the pyramid! Extent and quality of food advertised on Austrian television
Source: BMC Public Health. 2015 Sep 18;15:910. doi: 10.1186/s12889-015-2275-3 (PMC4574607; doi:10.1186/s12889-015-2275-3)
Supplement: Additional file 1: — Identified food products displayed in food advertisement, category and classification according the the EU Pledge Nutrition Criteria. (DOCX 101 kb) [file 12889_2015_2275_MOESM1_ESM.docx]

| **Identified Food (number of repetitions)** | **EU Pledge Category and Classification** |  | **EU Pledge Criteria** |
| --- | --- | --- | --- |
| Kinder Überraschung (38) ^≠^ | EU Pledge Category 5: Dairy products /Sub-category A: Sub-category A: Dairy Products other than cheeses: Must contain a minimum 50% dairy (Codex Alimentarius standard) |  | Not applicable^1^ |
| Kinder Schokolade (34) ^≠^ | EU Pledge Category 5: Dairy products /Sub-category A: Sub-category A: Dairy Products other than cheeses: Must contain a minimum 50% dairy (Codex Alimentarius standard) |  | Not applicable^2^ |
| Yo Fruchtsirup (26) ^≠^ | Not applicable^3^ |  | |
| Kinder Pingui (25) ^≠^ | EU Pledge Category 5: Dairy products /Sub-category A: Sub-category A: Dairy Products other than cheeses: Must contain a minimum 50% dairy (Codex Alimentarius standard) |  | Not applicable^4^ |
| Kinder Schoko Bons (21) ^≠^ | EU Pledge Category 5: Dairy products /Sub-category A: Sub-category A: Dairy Products other than cheeses: Must contain a minimum 50% dairy (Codex Alimentarius standard) |  | Not applicable^5^ |
| Haribo Goldbären (8) ^≠^ | Not applicable^6^ |  | |
| Stork Nimm 2 Soft (8) ^≠^ | Not applicable^6^ |  | |
| Stork Lachgummi (7) **^≠^** | Not applicable^6^ |  | |
| McDonald’s Dinner Box (4) **^≠^** | Not applicable^7^ |  | |

*Notes.* **^≠^** restricted according to the EU Pledge Criteria; ^1^ dairy amount in in the product < 50% (32%); ^2^ dairy amount in in the product < 50% (33%); ^3^ soft drink; ^4^ dairy amount in in the product < 50% (24%); ^5^ dairy amount in in the product < 50% (28%); ^6^ Sugar-based products (including chocolate or chocolate products, jam or marmalade, non-chocolate confectionery or other sugar products, sugar, honey or syrup); ^7^ Meals such as Hamburger, French Fries and Soft drink or even Mineral water; or any meal combination with Cheeseburger will not qualify.
