# Supplementary material for: Inverting the pyramid! Extent and quality of food advertised on Austrian television
Source: BMC Public Health. 2015 Sep 18;15:910. doi: 10.1186/s12889-015-2275-3 (PMC4574607; doi:10.1186/s12889-015-2275-3)
Supplement: Additional file 2: — Identified food products displayed in food advertisement, nutrient-based analysis according to the EU Pledge Nutrition Criteria. (DOCX 36 kb) [file 12889_2015_2275_MOESM2_ESM.docx]

| **Identified Food (number of repetitions)** | **EU Pledge Category**  **and Classification** | **EU Pledge**  **Criteria** | **Nutrient Content of the Product** | | |
| --- | --- | --- | --- | --- | --- |
| **Milchschnitte (48) ^≠^** | **EU Pledge Category 5: Dairy products / Sub-category A: Dairy Products other than cheeses: Must contain a minimum 50% dairy (Codex Alimentarius standard)** | | | | |
|  | Energy (kcal/portion) | ≤ 170 | 118 | | |
|  | Sodium (mg/100g) | ≤ 300 | 244 | | |
|  | Saturated fats (g/100g) | ≤ 2.6 | 16.6^*^ | | |
|  | Total sugars (g/100g) | ≤ 13.5 | 29.5^*^ | | |
|  | Components to encourage**^†^** | Protein >12 E% or > 2g /100g or 100ml  AND/OR at least 1 source of: Ca or Vit D or any Vitamin B | 7.9g Protein/100g | | |
| **Oreo (21) ^≠^** | **EU Pledge Category 6: Cereal based products / Sub-category A: Sweet biscuits, fine bakery wares and other cereal based products: cereal must be listed as the main ingredient on the ingredient declaration** | | | | |
| Energy (kcal/portion) | ≤ 200 | 135 | |  |  |
|  | Sodium (mg/100g) | ≤ 450 | 105 | |  |
|  | Saturated fats (g/100g) | ≤ 10 | 20^*^ | |  |
|  | Total sugars (g/100g) | ≤ 35 | 33.1 | |  |
|  | Components to encourage | Fibre (>3 g/100g) and/or whole grain  (15% total ingredients) and/or 20%E  from UFA and >70% UFA/total fat | 1.6g Fibre/100g | |  |
| **McDonald’s**  **Big Mac (17) ^≠^** | **EU Pledge Category 3: Meat based products: all kinds of processed meat/poultry, and meat products, consisting of minimally 50g of meat per 100g finished product** | | | |  |
|  | Energy (kcal/portion) | ≤ 170 | 509^*^ | |  |
|  | Sodium (mg/100g) | ≤ 800 | 430 | |  |
|  | Saturated fats (g/100g) | ≤ 6 | 5 | |  |
|  | Total sugars (g/100g) | ≤ 5 | 4 | | |
|  | Components to encourage | > 12% of energy as protein | 9.4% of energy as protein | | |
| **Hochland Ofen Aufstrich “Pizza” (8) ^≠^** | **EU Pledge Category 5: Dairy products / Sub-category A: Dairy Products other than cheeses: Must contain minimum 50% dairy (Codex Alimentarius standard)** | | | | |
|  | Energy (kcal/portion) | ≤ 170 | 231^*^ | | |
|  | Sodium (mg/100g) | ≤ 300 | 787^*^ | | |
|  | Saturated fats (g/100g) | ≤ 2.6 | 11.3^*^ | | |
| Total sugars (g/100g) | ≤ 13.5 | 3.8 | | |  |
| Components to encourage**^†^** | Protein >12 E% or > 2g /100g or 100ml  AND/OR at least 1 source of: Ca or Vit D or any Vit B | 12g Protein/100g | | |  |
| **Dr. Oetker Marmorette “Vanilla” (7) ^≠^** | EU Pledge Category 5: Dairy products / Sub-category A: Dairy Products other than cheeses: Must contain minimum 50% dairy (Codex Alimentarius standard) | | |  |  |
|  | Energy (kcal/portion) | ≤ 170 | 154 |  |  |
|  | Sodium (mg/100g) | ≤ 300 | 60 |  |  |
|  | Saturated fats (g/100g) | ≤ 2.6 | 5.6^*^ |  |  |
|  | Total sugars (g/100g) | ≤ 13.5 | 13.2 |  |  |
|  | Components to encourage**^†^** | Protein >12 E% or > 2g /100g or 100ml  AND/OR  At least 1 source of: Ca or Vit D or any Vit B | 3.1g Protein/100g |  |  |
| **Cheestrings (4)** | **EU Pledge Category 5: Dairy products / Sub-category B: Cheese and savoury dairy based products: Must contain minimum 50% dairy (Codex Alimentarius standard)** | | |  |  |
|  | Energy (kcal/portion) | ≤ 85 | 64 | | |
|  | Sodium (mg/100g) | ≤ 900 | 748 | | |
|  | Saturated fats (g/100g) | ≤ 15 | 14.2 | | |
|  | Total sugars (g/100g) | ˂ 5 | 1 | | |
|  | Components to encourage**^†^** | At least one source of: Ca, Vit B12,  Vit B2 | Calcium | | |
| **Dr. Oetker Pizza Tradizionale Mozarella (4) ^≠^** | **EU Pledge Category Category 7: Soups, composite dishes, main course and filled sandwiches / Sub-category B: Composite dishes, main dishes, and filled sandwiches** | | | | |
|  | Energy (kcal/portion) | ≤ 425 | 200 | | |
|  | Sodium (mg/100g) | ≤ 200 | 551^*^ | | |
|  | Saturated fats (g/100g) | ≤ 5 | 3 | | |
|  | Total sugars (g/100g) | ≤ 7.5 | 5.9 | | |
|  | Components to encourage | Nutrients delivered through  ingredients (fruits and/or vegetables,  cereals, meat, fish, milk) | n.d | | |
| **Danone**  **Actimel (4)** | **EU Pledge Category 5: Dairy products / Sub-category A: Dairy Products other than cheeses: Must contain minimum 50% dairy (Codex Alimentarius standard)** | | | | |
|  | Energy (kcal/portion) | ≤ 170 | 75 | | |
|  | Sodium (mg/100ml) | ≤ 300 | 39 | | |
|  | Saturated fats (g/100ml) | ≤ 2.6 | 1 | | |
|  | Total sugars (g/100ml) | ≤ 13.5 | 11.7 | | |
|  | Components to encourage **^†^** | Protein >12 E% or > 2g /100g or 100ml  AND/OR at least 1 source of: Ca or Vit D or any Vit B | 2.6g Protein/100g  Calcium, Vitamin D  Vitamin B | | |
| **Dr. Oetker Paula Stracciatella (3)** | **EU Pledge Category 5: Dairy products / Sub-category A: Dairy Products other than cheeses: Must contain minimum 50% dairy (Codex Alimentarius standard)** | | | | |
|  | Energy (kcal/portion) | ≤ 170 | 136 | | |
|  | Sodium (mg/100ml) | ≤ 300 | 79 | | |
|  | Saturated fats (g/100ml) | ≤ 2.6 | 2.4 | | |
|  | Total sugars (g/100ml) | ≤ 13.5 | 13 | | |
|  | Components to encourage **^†^** | Protein >12 E% or > 2g /100g or 100ml  AND/OR at least 1 source of: Ca or Vit D or any Vit B | 3g Protein/100g | | |
| **Müller Froop Safari Nektarine-Sternfrucht (2) ^≠^** | **EU Pledge Category 5: Dairy products / Sub-category A: Dairy Products other than cheeses: Must contain minimum 50% dairy (Codex Alimentarius standard)** | | | | |
|  | Energy (kcal/portion) | ≤ 170 | 155 | | |
|  | Sodium (mg/100g) | ≤ 300 | 130 | | |
|  | Saturated fats (g/100g) | ≤ 2.6 | 2 | | |
|  | Total sugars (g/100g) | ≤ 13.5 | 15* | | |
|  | Components to encourage **^†^** | Protein >12 E% or > 2g /100g or 100ml  AND/OR at least 1 source of: Ca or Vit D or any Vit B | 3.9g Protein/100g | | |
| **Dr. Oetker**  **Paula Pudding (1)** | **EU Pledge Category 5: Dairy products / Sub-category A: Dairy Products other than cheeses: Must contain minimum 50% dairy (Codex Alimentarius standard)** | | | | |
|  | Energy (kcal/portion) | ≤ 170 | 137 | | |
|  | Sodium (mg/100g) | ≤ 300 | 79 | | |
|  | Saturated fats (g/100g) | ≤ 2.6 | 2.5 | | |
|  | Total sugars (g/100g) | ≤ 13.5 | 13 | | |
|  | Components to encourage **^†^** | Protein >12 E% or > 2g /100g or 100ml  AND/OR at least 1 source of: Ca or Vit D or any Vit B | 3g Protein/100g | | |

*Notes.* **^≠^** restricted according to the EU Pledge Criteria; ^*^ values above threshold for allowed nutrients; **^†^** product contains components to encourage; n.d = no data available
